# Islet Specific Wnt Activation in Human Type II Diabetes

**DOI:** 10.1155/2008/728763

**Published:** 2009-01-20

**Authors:** Seung-Hee Lee, Carla Demeterco, Ifat Geron, Annelie Abrahamsson, Fred Levine, Pamela Itkin-Ansari

**Affiliations:** ^1^Development and Aging Program, Burnham Institute for Medical Research, 10901 N. Torrey Pines Road, La Jolla, CA 92037, USA; ^2^Department of Pediatrics, University of California San Diego, 9500 Gilman Dr, MC 0816, La Jolla, CA 92093, USA; ^3^Moores Cancer Center, University of California San Diego, La Jolla, CA 92093, USA; ^4^Sanford Children's Health Research Center, Burnham Institute for Medical Research, 10901 N. Torrey Pines Road, CA 92037, USA

## Abstract

The Wnt pathway effector gene TCF7L2 has been linked to type II diabetes, making it important to study the role of Wnt signaling in diabetes pathogenesis. We examined the expression of multiple Wnt pathway components in pancreases from normal individuals and type II diabetic individuals. Multiple members of the Wnt signaling pathway, including TCF7L2, Wnt2b, *β*-catenin, pGSK3*β*, TCF3, cyclinD1, and c-myc, were undetectable or expressed at low levels in islets from nondiabetic individuals, but were also upregulated specifically in islets of type II diabetic patients. Culture of pancreatic tissue and islet isolation led to Wnt activation that was reversed by the Wnt antagonist sFRP, demonstrating that Wnt activation in that setting was due to soluble Wnt factors. These data support a model in which the Wnt pathway plays a dynamic role in the pathogenesis of type II diabetes and suggest manipulation of Wnt signaling as a new approach to *β*-cell-directed diabetes therapy.

## 1. INTRODUCTION

The *β*-cell is a major target organ in the pathogenesis of type II diabetes as evidenced by the fact that overt diabetes does not occur until *β*-cell dysfunction/loss has proceeded to the point where hypersecretion of insulin can no longer compensate for peripheral insulin resistance [1]. The predominant model of the molecular events leading to *β*-cell failure is that the diabetic environment, in particular chronically high levels of glucose and free fatty acids, has toxic effects on the *β*-cell [2]. A number of signaling pathways have been implicated in *β*-cell failure, including insulin signaling [3] and oxidative stress [4].

Wnt signal transduction is one of the central pathways that control organismal growth and differentiation [5]. In the best-studied branch of the Wnt pathway, termed the canonical pathway, Wnts bind to frizzled receptors in conjunction with LRP family coreceptors. Resultant pathway activation prevents GSK3-mediated phosphorylation of *β*-catenin and its subsequent ubiquitin-mediated degradation. Stabilized *β*-catenin translocates into the nucleus where it interacts with transcription factors of the TCF family to activate target genes, including c-myc and cyclin D family members.

In addition to the canonical pathway, an alternative, noncanonical pathway with multiple branches exists. A calcium-dependent pathway acts through calcium/calmodulin-dependent kinase II (CamKII) primarily to control cell movement. In some cell types, activation of the noncanonical pathway leads to activation of the NFAT transcription factor [6], which plays an important role in the maintenance of functional *β*-cells [7].

Wnt signaling is critical in early foregut and pancreatic development [8–10]. Moreover, homozygous mutation of LRP5 in mice leads to defective glucose-stimulated insulin secretion from isolated islets in vitro [11]. Components of the Wnt pathway are present in the adult pancreas, and in particular multiple members of the frizzled family of Wnt receptors have been identified in the islet [8]. There are discrepant data on the role of Wnt signaling in mature islets. While most studies have found little evidence that Wnt signaling is involved in endocrine differentiation or in the adult islet [9, 10, 12], there is some evidence for an effect of Wnt signaling on *β*-cell replication [13].

To study the role of Wnt signaling in human diabetes, we systematically examined components of this pathway in the pancreas of normal and type II diabetic individuals. Strikingly, Wnt2b, which activates the canonical Wnt pathway, was highly upregulated in type II diabetes. Other mediators of the Wnt pathway, including pGSK3*β*, TCF/Lef factors, c-myc, and cyclinD1, were also induced in type II diabetes, specifically in the *β*-cells. *β*-catenin, in contrast to the mouse, in which it is highly expressed in normal islets [14], was low or absent in normal human islets in situ. However, it was highly upregulated in an islet-specific manner in human type II diabetes. Most *α*-cells did not exhibit Wnt pathway activation and instead expressed high levels of the noncanonical Wnt, Wnt4, which can act to repress canonical Wnt signaling [15]. The upregulation of TCF factors, including TCF7L2, is particularly interesting as linkage studies have identified a polymorphism in that gene as being tightly linked to type II diabetes in humans and there is some evidence that this polymorphism affects *β*-cell function [16–20].

Ectopic overexpression of the Wnt target gene c-myc in mice has been shown to cause *β*-cell apoptosis and diabetes [21]. To determine whether c-myc was a candidate for being an early effector in diabetes pathogenesis, we examined the effect of high-fat diet on c-myc expression in the murine pancreas. Interestingly, mice fed a high-fat diet for three months exhibited upregulated c-myc at a time when the mice were only mildly obese and not yet diabetic.

The finding that the Wnt pathway is activated in human type II diabetes and that the Wnt target gene c-myc is also upregulated early in progression to diabetes in rodents has important implications for understanding the mechanism of *β*-cell compensation and failure in diabetes and provides impetus for the development of new therapies targeted to that pathway.

## 2. METHODS

### 2.1. Tissue

Paraffin-embedded specimens from human pancreases (5 nondiabetic and 9 type II diabetic) were previously described [22]. Freshly isolated and cultured pancreases from three nondiabetic individuals were obtained from the Islet Resource center in (Seattle, Wash, USA) immediately prior to islet isolation. These pancreases were obtained postmortem by UNOS and shipped to Seattle in University of Wisconsin solution (UW solution). Specimens were collected in 4% paraformaldehyde (PFA) and shipped to us overnight followed by 30% sucrose/PBS, embedded in OCT freezing media (Sakura, Calif, USA) and snap-frozen at −80°C. Isolated human islets (cultured islets) were obtained through the Islet Procurement Resource Program (JDRF). Islets were shipped in CMRL 1066 medium with serum-free supplements. Upon arrival, islets were fixed in PFA or washed and cultured in suspension in RPMI supplemented with 5.5 mM glucose, penicillin (100 U/mL), streptomycin (100 ug/mL), and 10% bovine serum. Twenty four hours later, they were treated with 0, 100, 500 ng/mL rhsFRP-1(R&D systems, Minn, USA) for 48 hours, harvested by fixing in 4% neutral phosphate-buffered formalin, followed by 30% sucrose/PBS, embedded in OCT freezing media (Sakura, Calif, USA), and snap-frozen at −80°C.

Human fetal pancreases at 18–24 gestational weeks were obtained from Advanced Bioscience Resource, in accordance with University of California San Diego Institutional Review Board regulations (cold ischemia 12–24 hours).

Murine pancreases were obtained from anesthetized Balb/c or C57/bl mice and collected in 4% PFA followed by paraffin embedding.

Preparation of nonendocrine pancreatic cells (NEPCs) clusters was as described [23]. The unpurified islet fractions and Cobe bag fractions were obtained from the Universities of Edmonton, Minnesota, Miami, Washington, the Whittier Institute for Diabetes, and the City of Hope Medical Center.

### 2.2. Immuno-histochemistry

Both paraffin and frozen samples were sectioned to a mean thickness of 5 *μ*m, washed four times with PBS, treated with 0.3% Triton in PBS for 10 minutes, and incubated in blocking solution (PBS with 0.1% Triton X-100/5% normal goat serum) for 30 minutes at room temperature. They were then treated by alcohol series and antigen retrieval with CitriSolvTM (Fisher Scientific, Pa, USA). Sections were incubated in an antibody solution overnight at 4°C as described Table 1. For fluorescent imaging, slides were incubated with Alexa 488 (Molecular Probs, Ore, USA)/Rhodamine or Alexa 596 (Jackson Immuno Research, 1:300) fluor-labeled anti-Goat/mouse/rabbit for 45 minutes at room temperature. They were mounted in Vectashield (Vector Labs, Burlingame, Calif, USA). Digital images of stained sections were captured using a fluorescence microscope with a digital camera (Nikon, Tokyo, Japan) and deconvoluted (nearest neighbor) using SlideBook software (Intelligent Imaging Innovations, Denver, Colo, USA). Confocal microscopy was performed with an MRC 1024 MP laser scanning microscope (Bio-Rad Laboratories, Inc., Richmond, Calif, USA) equipped with krypton/argon laser and a Millenia-Tsunami two-photon Ti-Sapphire femtosecond laser system (Spectra-Physics, Mountain View, Calif, USA). Fluorescent intensity signals in confocal images from slides from nondiabetic and type II diabetic patients were scored blindly and quantitated using Image J histogram analysis. Statistical analysis was performed using Student's *t*-test to determine mean +/−SE.

### 2.3. Western blot

Whole-cell extracts were prepared by incubation in lysis buffer (50 mM Tris, pH 8, 150 mM NaCl, 5 mM EDTA, 100 mg/mL PMSF, 1 mg/mL aprotinin, 1% Triton X-100) for 30 minutes on ice, followed by 15 minutes of centrifugation at 12 000 g, 4°C as previously described [24]. Extracts were mixed with loading buffer and electrophoresed on 4–15% acrylamide gel (ReadyGel Bio-Rad Laboratories, Inc. Hercules, Calif, USA) in Tris/glycine/SDS buffer. Proteins were transferred onto double nitrocellulose membranes (Immobilon-Psq, Millipore, Bedford, Mass, USA) in Tris/glycine/methanol buffer for 1 hour at 100 V. After overnight blocking in PBS-Tween (PBST) with 3% milk, membrane was incubated for 2 hours at room temperature with antibodies: anti-*β*-catenin (Pharmingen), anti-CK19 (cytokeratin-19) (DAKO), anti-Actin (Sigma, St Louis, Mo, USA). After extensive washing in PBST, the membrane was incubated with secondary antibody conjugated to horseradish peroxidase diluted 1:2000 (Amersham/GE, Buckinghamshire, UK), washed in PBST, and revealed by ECL (Amersham/GE, Buckinghamshire, UK).

### 2.4. Mouse studies

Male C57BL/6J mice were purchased from Harlan Sprague Dawley, Inc. (Indianapolis, Ind, USA). The animals were obtained at 4 weeks of age weighing 17.9–21.2 g at the start of the study and kept four per cage on a 12:12-hour light dark schedule. The study was approved by the UCSD IACUC. One week after arrival, mice were divided into two groups and were fed either a high-fat diet (Research Diets, New Brunswick, NJ, USA) or received continuous feeding of a normal diet for up to 14 weeks. The high-fat diet consisted of 45% fat, 35% carbohydrate, and 20% protein (4.73 Kcal/g), whereas the normal diet contained 5% fat, 57% carbohydrate, and 18% protein (3.4 Kcal/g). A caveat to high-fat feeding experiments is the possibility that a high-fat diet differs from normal chow in multiple constituents, potentially confounding results [25].

For intraperitoneal glucose tolerance test (IPGTT), 8-hour fasted mice received an intraperitoneal injection with D-glucose (1.5 g/kg). Blood samples were obtained from the tail vein immediately before and at 15, 30, 60, 90, and 120 minutes after the glucose load. Blood glucose levels were measured with a Precision Xtra^ TM^ glucometer (Abbott Laboratories, Alameda, Calif, USA).

## 3. RESULTS

### 3.1. TCF7L2 is upregulated in islets of type II diabetic patients

**Figure 1:**
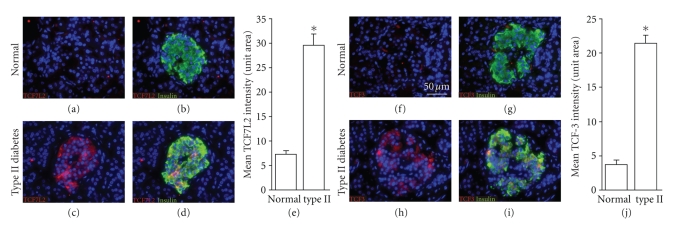
*Expression of TCF factors in type II diabetes*. TCF7L2 (red in (a)–(d)) and TCF3 (red in (f)–(i)) are absent from islets of nondiabetic individuals (a), (b), (f), and (g), but are present in islets from type II diabetics (c), (d), (h), and (i). Islets are identified by insulin (green in (b), (d), (g), and (i)). Quantitation of TCF7L2 (e) and TCF3 (j) expressions in nondiabetic (*n* = 4) and type II (*n* = 7) islets: error bars = mean +/− s.e.m. *P < .05. Scale bars = 50 *μ*m.

Recently, TCF7L2 has been genetically linked to type II diabetes in multiple populations [16–19]. However, the mechanism by which it contributes to diabetes pathogenesis is unknown. The mRNA level of TCF7L2 is higher in cultured isolated islets from type II patients versus normal individuals [20], but expression in situ has not been examined. We found that TCF7L2 protein was barely detectable in the pancreas of normal individuals, but it was highly upregulated in an islet-specific manner in patients with type II diabetes (Figures1(a)–1(e)). In addition, the expression of TCF3, which has not been linked to type II diabetes, was also upregulated (Figures 1(f)–1(j)).

### 3.2. Wnt2b is upregulated in type II diabetes

**Figure 2:**
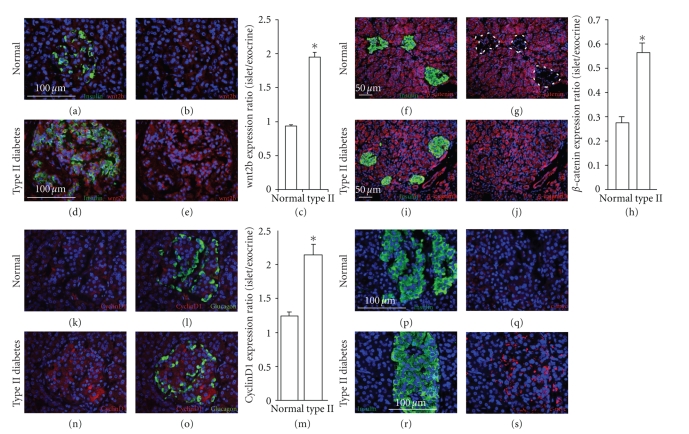
*Expression of Wnt2b, *β*-catenin, cyclinD1, and c-myc in type II diabetes*. Wnt2b (red in (a), (b), (d), and (e)), *β*-catenin (red in (f), (g), (i), and (j)), cyclinD1 (red in (k), (l), (n), and (o)), and c-myc (red in (q) and (s)) are absent from islets of nondiabetic individuals (a), (b), (f), (g), (k), (l), (p) and (q), but are present in islets from type II diabetics (d), (e), (i), (j), (n), (o), (r), and (s). Islets are identified by insulin (green in (a), (d), (f), (i), (p), and (r)) or glucagon (l) and (o) and are outlined by a dashed line in (g) to better demonstrate the absence of *β*-catenin. Quantitation of Wnt2b (c), *β*-catenin (h), and cyclinD1 (m) expression in non-diabetic (*n* = 5) and type II (*n* = 7) islets: error bars = mean +/− s.e.m. *P < .05. Scale bars = 100 *μ*m except for f.g.i.j which are 50 *μ*m.

Because TCF7L2 is both an effector as well as a downstream target of Wnt signaling [26], the induction of TCF factors suggested that a more global activation of Wnt signaling may be occurring. To test that hypothesis, we examined the expression of soluble Wnt factors and the initiators of Wnt signaling. Based upon the reported pattern of expression of soluble Wnt factor mRNA in the pancreas, we examined Wnt2b, which was a good candidate as its RNA has been reported to be present in isolated islets [8, 27]. In nondiabetic individuals, little or no Wnt2b expression was detectable (Figures 2(a), 2(b), 2(c)). However, there was dramatic upregulation in the islets of type II diabetics. Robust expression of Wnt2b occurred in both *β*-cells (Figures 2(c), 2(d), 2(e)) and *α*-cells (not shown) of diabetic patients.

### 3.3. Human *β*-cells lack detectable *β*-catenin expression but it is strongly upregulated in type II diabetes

Activation of frizzled receptors by soluble Wnts results in the stabilization of *β*-catenin. *β*-catenin in human islets of all five nondiabetic individuals examined was markedly lower than in the surrounding exocrine tissue, where it was strongly expressed (Figures 2(f)–2(h), Supplementary Figures 1(a), 1(b) available online at doi:10.1155/2008/728763 ). The same pattern was observed in the human fetal pancreas, with high levels of *β*-catenin expression in nonendocrine epithelial cells and very low levels in endocrine cells (Supplementary Figures 1(c), 1(d)). Colocalization studies with PDX-1 revealed that *β*-catenin was substantially restricted to nonendocrine PDX-1 positive, cells in the human fetal pancreas (Supplementary Figure 1(e)). Because PDX-1 serves as a precursor for both exocrine and endocrine compartments, *β*-catenin expression must be repressed during human, but not murine, endocrine differentiation.

To determine whether *β*-catenin plays a role in *β*-cell dysfunction and/or loss in human diabetes, its expression was examined in the pancreases of 9 patients with type II diabetes. In contrast with nondiabetic pancreases (Figures 2(f)–2(h)), it was strongly expressed in the islets of all 9 patients, to a level approximately half that of the surrounding exocrine tissue (Figures 2(h)–2(j)). In contrast, there was no noticeable effect of type II diabetes on the expression of E-cadherin, which is the major islet cadherin and may also influence the level of *β*-catenin protein in the cell by sequestering it from degradation (not shown).

Because *β*-catenin is regulated by GSK3*β*, we examined the state of GSK3*β* activation in normal and type II diabetic pancreases. The inactive, phosphorylated form of GSK3*β*, pGSK3*β*, that leads to stabilization of *β*-catenin, was limited to islets in both nondiabetic and diabetic individuals (Supplementary Figure 2). In type II diabetics, 95% of islets expressed pGSK3*β*, while 80% of islets from nondiabetic individuals expressed pGSK3*β*. Thus, the majority of normal islets expressed pGSK3*β* despite having low or undetectable levels of *β*-catenin, suggesting that the upregulation of *β*-catenin in islets is not controlled in a simple way by the level of pGSK3*β*.

### 3.4. *γ*-catenin is expressed in human *β*-, but not *α*-, cells

The low level of *β*-catenin in normal human islets in vivo prompted us to investigate whether another molecule might be substituting for it in mediating its signaling and/or structural roles in the *β*-cell. Lower organisms such as Drosophila have a single cadherin-binding catenin, Armadillo. However, in mammals, there are two such catenins, *β*- and *γ*-catenin, the latter also called plakoglobin [28]. In murine *β*-cells, *γ*-catenin was upregulated when *β*-catenin was deleted, but this was not noted to have any functional significance [10]. In the adult human pancreas, we found that *γ*-catenin was expressed in a highly selective manner in *β*-cells (Supplementary Figures 1(f)–1(h)), in contrast to *α*-cells and the surrounding exocrine tissue, where it was low or undetectable (Supplementary Figures 1(i)–1(k)). Thus, *α*-cells do not appear to exhibit high level expression of either catenin.

The pattern of catenin expression in the adult was mirrored in the human fetal pancreas, with *γ*-catenin being expressed in *β*-, but not *α*-cells (Supplementary Figures 1(l)–1(q)). Interestingly, in the human fetal pancreas, *γ*-catenin was also expressed in nonendocrine PDX-1 positive cells, being absent in PDX-1 negative cells (Supplementary Figures 1(r)–1(u)). Thus, in the human fetal pancreas, *β*- and *γ*-catenin are both expressed in PDX-1 expressing progenitors, but are inversely regulated as those progenitors diverge to form mature exocrine and *β*-cells.

### 3.5. Terminal effectors of Wnt signaling are upregulated in human type II diabetes

TCF/LEF factors activate a number of terminal effectors of Wnt signaling. A well-studied member of that group is c-myc [29]. Strikingly, c-myc was expressed in the islets from pancreases from patients with type II diabetes, but not in islets from nondiabetic individuals (Figures 2(p)–2(s)). Despite the role of c-myc in promoting cell proliferation, no increase in islet cell proliferation was observed, as measured by Ki67 staining (data not shown). In addition to c-myc, cyclinD1 is an important regulator of proliferation that is induced by Wnt signaling [30]. It was also highly upregulated in the islets of type II diabetic patients (Figures 2(k)–2(o)).

### 3.6. Wnt target expression is limited to *β*-cells, with *α*-cells being unaffected

**Figure 3:**
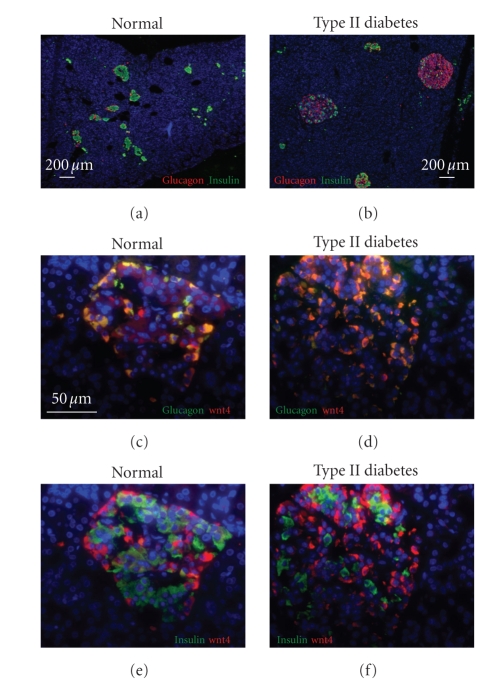
**α*-cell hyperplasia in type II diabetes and expression of Wnt4 in *α*-cells*. Immunostaining of pancreas from nondiabetic (a), (c), and (e) and type II diabetic (b), (d), and (f) individuals demonstrates *α*-cell hyperplasia in type II diabetes (a) and (b) and expression of Wnt4 (red in (c)–(f)) predominantly in *α*-cells (c)–(f). Scale bar in (a) and (b) = 200 *μ*m and in (c)–(f) = 50 *μ*m.

While upregulation of Wnt2b occurred throughout the islet, activation of Wnt downstream signaling was limited to *β*-cells, with *α*-cells in type II diabetic islets lacking expression of activation markers, including cyclinD1 (Figures 2(m)–2(o)). The lack of Wnt signaling in *α*-cells is interesting, as significant *α*-cell hyperplasia was observed in the type II diabetic pancreases (Figures 3(a), 3(b)) and is consistent with previous reports of the effect of type II diabetes on the endocrine pancreas [31].

Mechanistically, the fact that Wnt2b was upregulated in *α*-cells, but downstream signaling was not activated, suggested that the pathway is defective or repressed in those cells. Occasional *α*-cells expressing a low level of cyclinD1 and c-myc was more consistent with a repressor being present than with *α*-cells having a completely defective pathway. Members of the Wnt family that mediate noncanonical Wnt signaling repress signaling through the canonical pathway under some circumstances [15]. Thus, we studied the expression of the noncanonical Wnt, Wnt4, which microarray studies had suggested was expressed at a high level in human islets (unpublished). Consistent with the model, Wnt4 was expressed in *α*-, but not *β*-cells (Figures 3(c)–3(f)).

### 3.7. Islet isolation mimics the effect of type II diabetes on *β*-catenin expression

**Figure 4:**
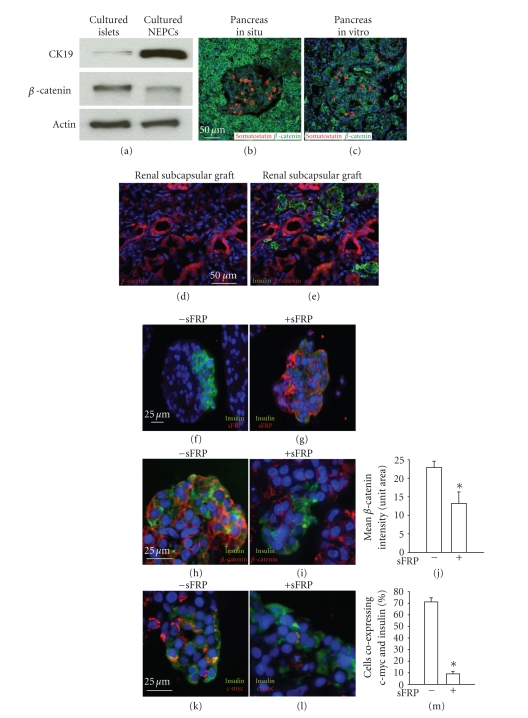
*Wnt signaling is induced in islets in vitro*. Western blot analysis (a) indicates that *β*-catenin is expressed in both cultured purified islets and cultured purified nonendocrine pancreatic clusters (NEPCs). Islets were dithizone picked to 99% purity, verified by qPCR as previously reported (23). As expected, the duct marker CK 19 is expressed at higher levels in NEPCs than in purified islets. Actin expression confirmed equivalent sample loading. (b) and (c) *β*-catenin (green in (b) and (c)) and somatostatin (red to visualize islets) staining demonstrate a low or absent *β*-catenin expression in islets of pancreatic tissue removed and fixed immediately postmortem (b) compared with high *β*-catenin expression in islets of pancreas tissue removed and fixed after the entire pancreas was shipped using the bilayer method to an islet isolation center (c). *β*-catenin is downregulated in cultured human islets following transplantation under the kidney capsule of Scid mice (d) and (e). Scale bars for (b)–(e): 50 *μ*m. Islets isolated from nondiabetic individuals (*n* = 3) were cultured for 48 hours in the absence (f), (h), and (k) or presence (g), (i), and (l) of 500 ng/mL of the Wnt inhibitor sFRP. Immunohistochemical analysis of insulin (green) and anti-sFRP (red in (f) and (g)) detects sFRP on islet cells only when sFRP has been added to the culture media. sFRP exposure led to inhibition of *β*-catenin ((h) versus (i), quantitated in (j)) and c-myc ((k) versus (l), quantitated in (m)). Error bars = mean +/− s.e.m. *P < .05. Scale bars in (f)–(m): 25 *μ*m.

To pursue the role of Wnt activation in the islet, it would be desirable to have an in vitro model. Thus, we examined Wnt activation in isolated islets. Surprisingly, when cultured human islets were examined by Western blotting, *β*-catenin, which is low or absent in the islet compared with surrounding tissue in situ, was expressed at a higher level than in the nonendocrine pancreatic cells (NEPCs) [23] (Figure 4(a)). To determine whether this was due to the process of islet isolation or subsequent in vitro culture in medium containing fetal bovine serum, we examined fragments from whole pancreas that were fixed following transport using the bilayer method to the islet isolation center [32], but prior to islet isolation itself. In those fragments, *β*-catenin induction occurred to a similar extent as in isolated islets (cf. Figures 4(b), 4(c)) marking it as an early event in the tissue procurement process. To determine whether upregulation of *β*-catenin in isolated islets indicated active canonical Wnt signaling, we examined them for c-myc expression, finding that it was highly expressed (Figures 4(k), 4(m)).

### 3.8. The Wnt inhibitor sFRP reduces *β*-catenin and c-myc expression in human islets

The model suggested by the data in type II diabetes and in isolated islets is that upregulation of soluble Wnts by some aspect of the environment in type II diabetic individuals or in isolated islets then activates the Wnt signaling pathway. However, alternative mechanisms for Wnt pathway activation are possible as well. For example, insulin, which is hypersecreted at early stages in type II diabetes in response to end organ unresponsiveness, is a potent inhibitor of GSK3 and thus a potential activator of the Wnt pathway [33].

To distinguish between Wnt-dependent and Wnt-independent activation of downstream signaling, human islets were cultured with or without soluble frizzled receptor protein (sFRP), which represses Wnt signaling by sequestration of Wnt proteins [34]. sFRP bound at high levels to islets (Figure 4(g)). Consistent with canonical activation of the pathway by secreted Wnts interacting with frizzled receptors, sFRP caused a reduction in both cytoplasmic and membrane-associated *β*-catenin (Figures 4(h)–4(j)) and c-myc (Figures 4(k)–4(m)). Thus, a Wnt inhibitor restored human beta cells to the *β*-catenin and c-myc negative state associated with normal tissue in situ, demonstrating clearly that Wnt pathway activation is occurring and suggesting that the process of Wnt activation in isolated islets mimics what occurs in type II diabetes.

Activation of Wnt signaling in harvested islets has implications for islet transplantation. To determine whether Wnt activation was reversible following transplantation, three cultured islet preparations (containing some nonendocrine material) were transplanted under the kidney capsule of immunodeficient mice and analyzed three months later. *β*-catenin expression in the islets had reverted back to levels much lower than in the surrounding duct structures within the graft (Figures 4(d), 4(e)), similar to the pattern found in the normal human pancreas.

### 3.9. Wnt effectors are inversely correlated with insulin expression in type II diabetes

**Figure 5:**
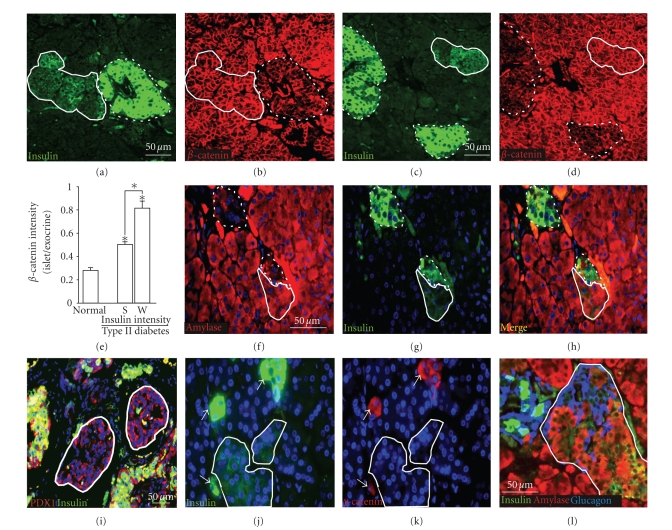
**β*-catenin is upregulated in islets from type II diabetic patients*. Insulin (a) and (c) and *β*-catenin (b) and (d) are inverely expressed in the pancreas of type II diabetic patients (a)–(d). Islets with weak insulin expression (solid lines) had the highest levels of *β*-catenin and islets with strong insulin expression (dashed lines) had levels of *β*-catenin intermediate between the weak insulin expressing islets and islets from nondiabetic individuals. Quantitation of *β*-catenin expression in *β*-cells from nondiabetic and type II diabetic individuals with strong (S) and weak (W) insulin staining (e). Insulin staining of weak islets was 3.5-fold less intense than in islets with strong insulin staining. More than 200 islets were examined (*P* < .05). Amylase (red in (f)) and insulin (green in (g)) colocalized in weak insulin-expressing islets in type II diabetes (solid line) but not in high insulin expressing islets (dashed line) (merged in (h)). Weak insulin expressing islets (marked with solid lines) retained PDX-1 expression (red in (i)) but lost *γ*-catenin ((j) and (k), islet with strong insulin marked with arrowheads). Weak insulin expressing islets (solid line) contained normal glucagon expressing cells that did not express amylase (l). Scale bars: 50 *μ*m. All images are confocal.

In two pancreases from patients with type II diabetes but none of the nondiabetic controls, there were two distinct types of islets, exhibiting a 3.5-fold difference in the level of insulin expression in the *β*-cells. Interestingly, *β*-catenin upregulation was present to a much greater degree in *β*-cells with diminished insulin expression (Figures 5(a)–5(e)). This indicates either that local factors are generated as a result of the diabetic environment or that islets are heterogeneous, with some being more susceptible to effects of factors that are homogeneously distributed.

Since *γ*- and *β*-catenin were inversely regulated in the normal pancreas, the upregulation in some type II diabetic pancreases of *β*-catenin in islets with lower insulin expression suggested the possibility that *γ*-catenin expression might be downregulated in those islets. This proved to be true (Figures 5(j), 5(k)). Thus, the weak insulin-expressing islets present in the diabetic state mimicked the pattern of catenin expression in the exocrine pancreas, where *β*-catenin protein is abundant and *γ*-catenin is not expressed.

To pursue the finding that *β*-cells with low-insulin expression had a pattern of catenin expression resembling that in the exocrine pancreas, pancreas sections were immunostained for the acinar marker amylase as well as insulin, revealing that low-insulin *β*-cells coexpressed amylase (Figures 5(f), 5(g), 5(h)). The insulin/amylase double-positive cells expressed PDX-1 (Figure 5(i)), which in the adult pancreas is restricted to *β*-cells and is never expressed in mature acinar cells, indicating that the weak insulin expression was not artifactual. The finding of cells expressing both insulin and amylase is consistent with a proposal by some investigators that endocrine and exocrine cells can transdifferentiate [35]. Insulin-amylase double-positive cells have been found in models of *β*-cell regeneration [36–38].

To further explore whether the areas containing the insulin/amylase double-positive cells arose by alteration of preexisting *β*-cells or by induction of insulin expression in preexisting exocrine cells, as has been described in some *β*-cell regeneration models [37–39], we examined those areas for glucagon expression. Consistently, high levels of glucagon and a lack of amylase were observed in all *α*-cells, whether the islets exhibited high or low insulin expression (Figure 5(l)). Thus, the *α*-cells appeared normal, even in low-insulin expressing islets. Overall, these data suggest that one effect of type II diabetes on the *β*-cell is to promote an aberrant differentiation state in which *β*-cells lose insulin expression and begin to express exocrine markers.

### 3.10. Expression of the Wnt target gene c-myc is an early response to high-fat diet

**Figure 6:**
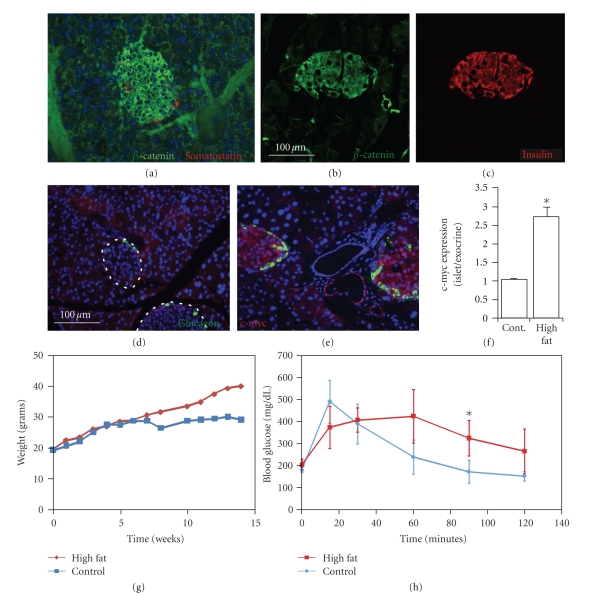
*Wnt signaling in high-fat fed mice*. In normal mouse pancreas, *β*-catenin (green in (a) and (b)) is expressed in islets as identified by somatostatin (red in (a)) and colocalizes with insulin (red in (c)). c-myc (red in (d) and (e)) was not expressed in islets of normal mice (marked by dotted lines and glucagon in green in (d)) but was induced in islets and some ducts of high-fat fed mice (islets marked by glucagon in green). C-myc expression is quantitated in (f). At 12 weeks, the time of analysis, high-fat mice were obese (g), and nondiabetic but mildly glucose-intolerant as measured by IPGTT (h). Number of normal mice = 3 and number of high-fat mice = 4 for (f), (g), and (h). Error bars = mean +/− s.e.m. *P < .05. Scale bars: 100 *μ*m.

To begin to address the question of whether Wnt activation was an early or late event in diabetes pathogenesis, we moved to a mouse model. Mice were placed on either a normal chow (low-fat) or high-fat diet and harvested pancreases were examined for c-myc expression after 12 weeks of high-fat diet, at which point the mice were obese (Figure 6(g)), but had a normal fasting blood glucose. Intraperitoneal glucose tolerance tests revealed mild glucose intolerance, being marginally statistically different from the control group only at the 90-minute time point (*P* = .04) (Figure 6(h)).

Consistent with previous studies [14], we found that the murine pancreas exhibited a pattern of *β*-catenin expression opposite from that in the human, with mouse islets expressing high levels of *β*-catenin and the exocrine pancreas having less *β*-catenin (Figures 6(a)–6(c)). However, the high level of *β*-catenin expression even in the normal chow group did not reflect Wnt activation, as c-myc was not detectable in either the endocrine or exocrine pancreas (Figures 6(d), 6(f)). In contrast, the high-fat fed animals exhibited a high level of c-myc in islets (Figures 6(e), 6(f)). Additionally, some but not all ducts in the high-fat fed animals exhibited c-myc expression (Figure 6(e)). Unlike in the human, where we were able to identify Wnt2b and Wnt4 as being expressed in the islet, we have thus far been unable to identify the soluble Wnts responsible for activating Wnt targets in the adult mouse pancreas. However, combined with the human data, the results in the mouse suggest that obesity alone may be sufficient to induce Wnt activation, which would mark it as an early event in the pathogenesis of type II diabetes.

## 4. DISCUSSION

The studies presented here are based primarily upon the examination of pancreas samples from normal and type II diabetic humans, from which we conclude that Wnt signaling effectors are upregulated in the islets in type II diabetes.

A number of components in the Wnt pathway have been found to be associated with type II diabetes or obesity in linkage studies. Our finding that TCF3 and TCF7L2 are induced in islets from type II diabetics is particularly interesting in light of recent linkage association studies in which TCF7L2 has been identified as the gene most strongly linked to type II diabetes [17–19]. TCF7L2 is also a direct downstream target of *β*-catenin [26], which we found to be induced in type II diabetes. Similarly, lrp5, a component of the Wnt receptor signaling complex is associated with obesity phenotypes [40] and Wnt5b is associated with susceptibility to type II diabetes [41]. Wnt pathway genes have also been linked to type I diabetes [42]. Whether the involvement of Wnt signaling on predisposition to diabetes occurs through effects in the islet or in peripheral tissue has not been determined. However, we and others have established that c-myc has substantial effects on *β*-cell function, exemplified by its ability to repress hormone expression and to induce both proliferation and apoptosis [43, 44].

Of relevance to the specificity of the changes in Wnt signaling that were observed, alterations in the level of Wnt pathway components in the human pancreas were restricted to the islet, with little change in the exocrine pancreas. Consistent changes in Wnt pathway components were evident despite the fact that the human pancreases were obtained from widely divergent patients. The “normal” controls were mostly not from young healthy individuals, but rather from older individuals with significant illness [22]. This is likely to have minimized the extent of any differences, so it is striking that we still observed large changes in the expression of Wnt pathway components in the samples from diabetic patients.

While no single Wnt downstream effector or pathway intermediate is absolutely specific for the Wnt pathway given the extensive cross-talk between Wnt and other signaling pathways, the evidence for activation of Wnt signaling in *β*-cells is strong, with pathway components at all levels being affected, including upregulation of *β*-catenin, TCF3, TCF7L2, cyclinD1, c-myc, and Wnt2b. Upregulation of Wnt2b is important, as there cannot be true pathway activation in the absence of an initiating signal. The downregulation of the downstream target c-myc by sFRP provides direct evidence that Wnt activation through a soluble Wnt occurs in islets, though the stimulus for upregulation of the soluble Wnt may or may not be the same in isolated islets and type II diabetes.

While Wnt2b was induced throughout the islet, canonical Wnt activation was found predominantly in *β*-cells. In *α*-cells, the pathway remained inactive, a state that correlated with *α*-cell-specific expression of Wnt4, a member of the Wnt family that mediates noncanonical signaling and that can repress canonical signaling [15, 45]. While we observed changes in expression of Wnt2b and Wnt4, the Wnt family is large. Attempts to examine other members are ongoing but have been limited by the lack of high-quality antibodies. It is likely that additional complexity will be revealed as other members of the Wnt family and other components of the pathway are examined.

Wnt binding to frizzled receptors leads to stabilization of *β*-catenin by inactivation of GSK3*β*, which is itself under complex control, including distinct regulation by growth factors as well as Wnt signaling [46]. Of relevance to diabetes, GSK3*β* is inhibited by insulin signaling through the PI3 kinase pathway [47]. Thus, hyperinsulinemia, a classic feature of type II diabetes that occurs early in the disease, could lead quite directly to upregulation of *β*-catenin. Interestingly, it has been proposed, on the basis of its involvement in insulin signaling, that inhibitors of GSK3 may be effective in improving insulin sensitivity in rodent models of type II diabetes [48]. However, based on data presented here, such inhibitors might lead to Wnt activation in the islet. The inverse correlation between Wnt pathway activation and insulin gene expression that we found suggests that, at least in the long term, Wnt activation could be deleterious to islet function. In contrast to what is believed to occur in the peripheral tissue, it is possible that Wnt inhibition and consequent activation of GSK3*β* could have positive effects on islet function. Thus, in one tissue, GSK3 inhibition might prove beneficial, while in another, harmful.

*β*-cell specific Wnt effector upregulation occurred in the context of striking differential expression of Wnt pathway components in the endocrine versus exocrine pancreas in nondiabetic humans and mice, as well as significant interspecies differences. The best example of this is *β*-catenin, which was expressed in an inverse pattern in the normal human and murine pancreases. In humans, *β*-catenin was virtually absent in islets and expressed at high levels in the exocrine pancreas, while in the murine pancreas the reverse pattern was found. In the human exocrine pancreas, *β*-catenin appears to be localized to the plasma membrane, which is a pattern that is consistent with its role in mediating connections between cadherins and the actin cytoskeleton. Despite decreased *β*-catenin expression in islets, only the human endocrine pancreas expressed high levels of phosphorylated GSK3*β*, which in the canonical model of Wnt signaling should have led to stabilization and upregulation of *β*-catenin. Unfortunately, we were unable to assess the expression of the unphosphorylated, active form of GSK3*β* due to limitations of the available antibodies.

*γ*-catenin was expressed in a converse fashion to *β*-catenin in islets from nondiabetic individuals. Further, the dynamic regulation of *β*- and *γ*-catenin, with *β*-catenin being induced and *γ*-catenin repressed in islets with activated Wnt signaling, suggests that these molecules play an important role in diabetes pathogenesis. In some tissues, *β*- and *γ*-catenin act antagonistically, with *β*-catenin tending to promote cell growth, while *γ*-catenin acts as a tumor suppressor [49, 50]. While both *β*- and *γ*-catenin can bind to TCF/LEF transcription factors, *γ*-catenin does so much less efficiently [51]. In addition, we have found that TCF genes are specifically activated by *β*-, but not *γ*-catenin (unpublished results).

Wnt pathway activation in isolated islets has implications for islet transplantation. Islet dysfunction and loss is a major problem in islet transplantation, both in the immediate posttransplant period and chronically [52]. The finding that the process of isolation induces some changes that are also seen in type II diabetes raises the possibility that the isolation process could have deleterious effects. If, as indicated by the known undesirable effects of c-myc on islet biology [43, 44, 53], Wnt activation has a negative effect on *β*-cell function and/or survival, inhibiting that upregulation in transplanted islets could be beneficial. The demonstration here that *β*-catenin upregulation is reversible following renal subcapsular transplantation or by inhibiting Wnt signaling with sFRP provides a direct means of testing that hypothesis and could also provide a model system to study the effects of Wnt pathway activation in type II diabetes.

A question of major importance is the functional role of Wnt signaling in diabetes pathogenesis, that is, is it part of an adaptive response or a pathologic response? These are not mutually exclusive. One possibility is that Wnt activation plays an adaptive role early in type II diabetes, perhaps in promoting *β*-cell proliferation [13], while chronic pathway activation leads to cell death, a well-recognized function of c-myc [44, 54]. The mouse studies suggest but do not yet prove definitively that Wnt activation could be an early event in diabetes pathogenesis that is associated with high-fat diet.

The striking activation of Wnt signaling in human type II diabetes, albeit in a small number of patients, provides insight into the molecular effects of diabetes on the *β*-cell and offers a novel potential route to prevent *β*-cell failure. Direct manipulation of the activation state of the pathway is required to dissect out the complex roles of the Wnt pathway in diabetes. In addition, diabetes has been shown to be a strong risk factor for cancer, including pancreatic cancer [55, 56]. Moreover, overexpression of *β*-catenin in the mouse pancreas promoted pancreatic cancer [57]. Thus, activated Wnt signaling may be a critical mechanism of pathogenesis common to both diabetes and pancreatic cancer.

## Supplementary Material

The inverse expression pattern of *γ*- and ß-catenin in normal adult and fetal human pancreas. Supplementary Figure 2 illustrates the expression pattern of pGSK3ß in the normal and Type II pancreas, revealing that there is higher expression in Type II than in normal islets.

## Figures and Tables

**Table 1:** Antibodies.

Antibody	Cat. No.	Supplier	Conc.
Amylase	SC-12821	Santa Cruz	1:300
*β*-catenin	SC-7963	Santa Cruz	1:200
*β*-catenin	SC-7199	Santa Cruz	1:200
*β*-catenin	610154	Pharmingen	1:200
c-myc	554205	Pharmingen	1:200
c-myc	NCL-CMYC	Novocastra	1:100
Cyclin D1	SC-718	Santa Cruz	1:200
E-cadherin	SC-7870	Santa Cruz	1:200
*γ*-catenin	SC-8415	Santa Cruz	1:200
*γ*-catenin	SC-7900	Santa Cruz	1:200
Glucagon	G-2654	Sigma	1:1000
Glucagon	AB932	Chemicon	1:500
Insulin	SC-8033	Santa Cruz	1:200
Insulin	SC-9168	Santa Cruz	1:200
Insulin	I7660	US Biological	1:200
Pdx-1		Dr. Chris Wright	1:1000
pGSK3*β*	SC-11757	Santa Cruz	1:200
sFRP-1	AF1384	R&D	1:200
Somatostatin	SC-13099	Santa Cruz	1:200
TCF-3	SC-8635	Santa Cruz	1:200
TCF7L2	SC-8631	Santa Cruz	1:200
Wnt2b	AF3900	R&D	1:100
Wnt4	AF475	R&D	1:100
