# Supplementary figures and images for: Islet Specific Wnt Activation in Human Type II Diabetes
Source: Exp Diabetes Res. 2009 Jan 20;2008:728763. doi: 10.1155/2008/728763 (PMC2628766; doi:10.1155/2008/728763)

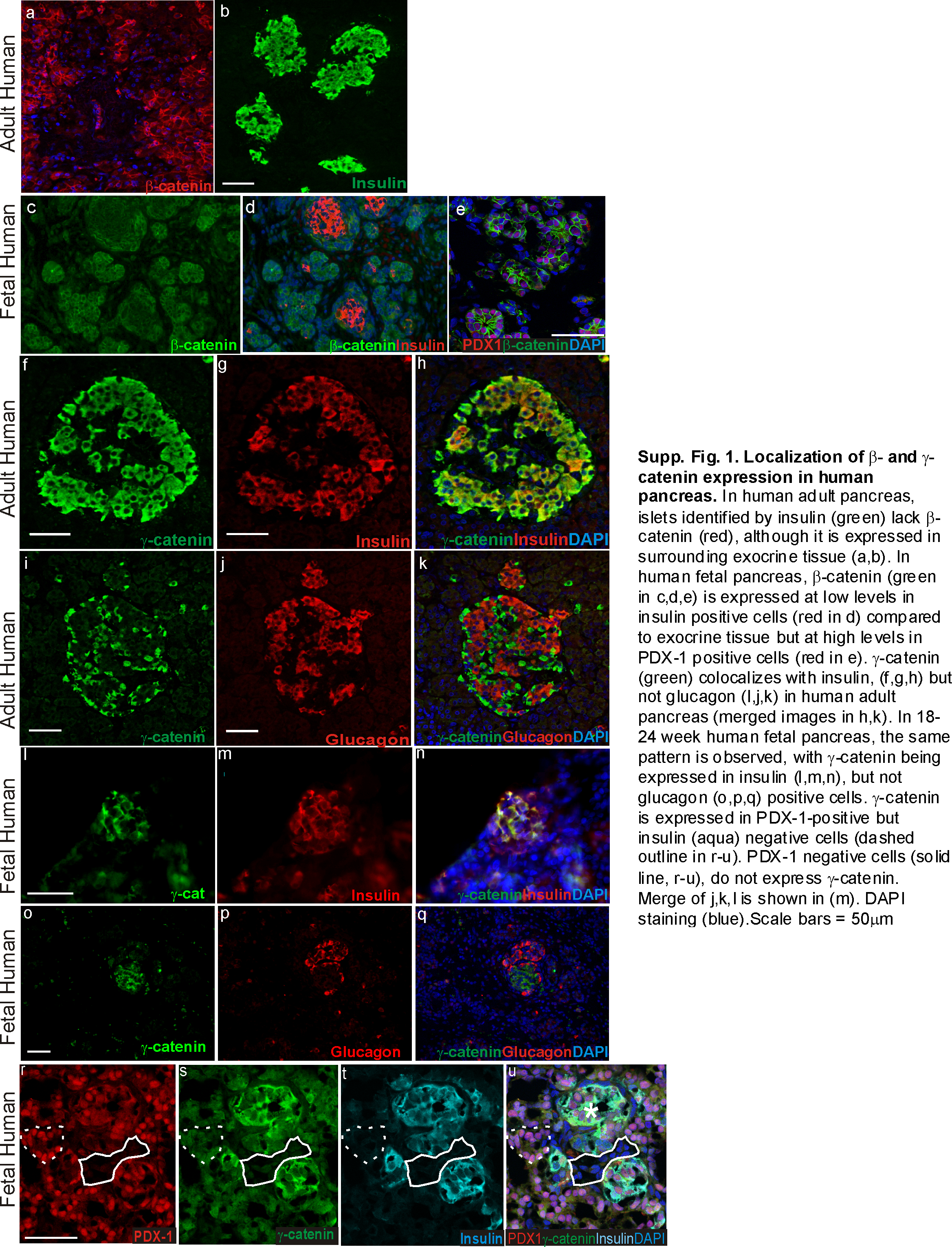

Supplement: Supplementary file 1 — The inverse expression pattern of γ- and ß-catenin in normal adult and fetal human pancreas. Supplementary Figure 2 illustrates the expression pattern of pGSK3ß in the normal and Type II pancreas, revealing that there is higher expression in Type II than in normal islets. [file 728763.f1.tif]

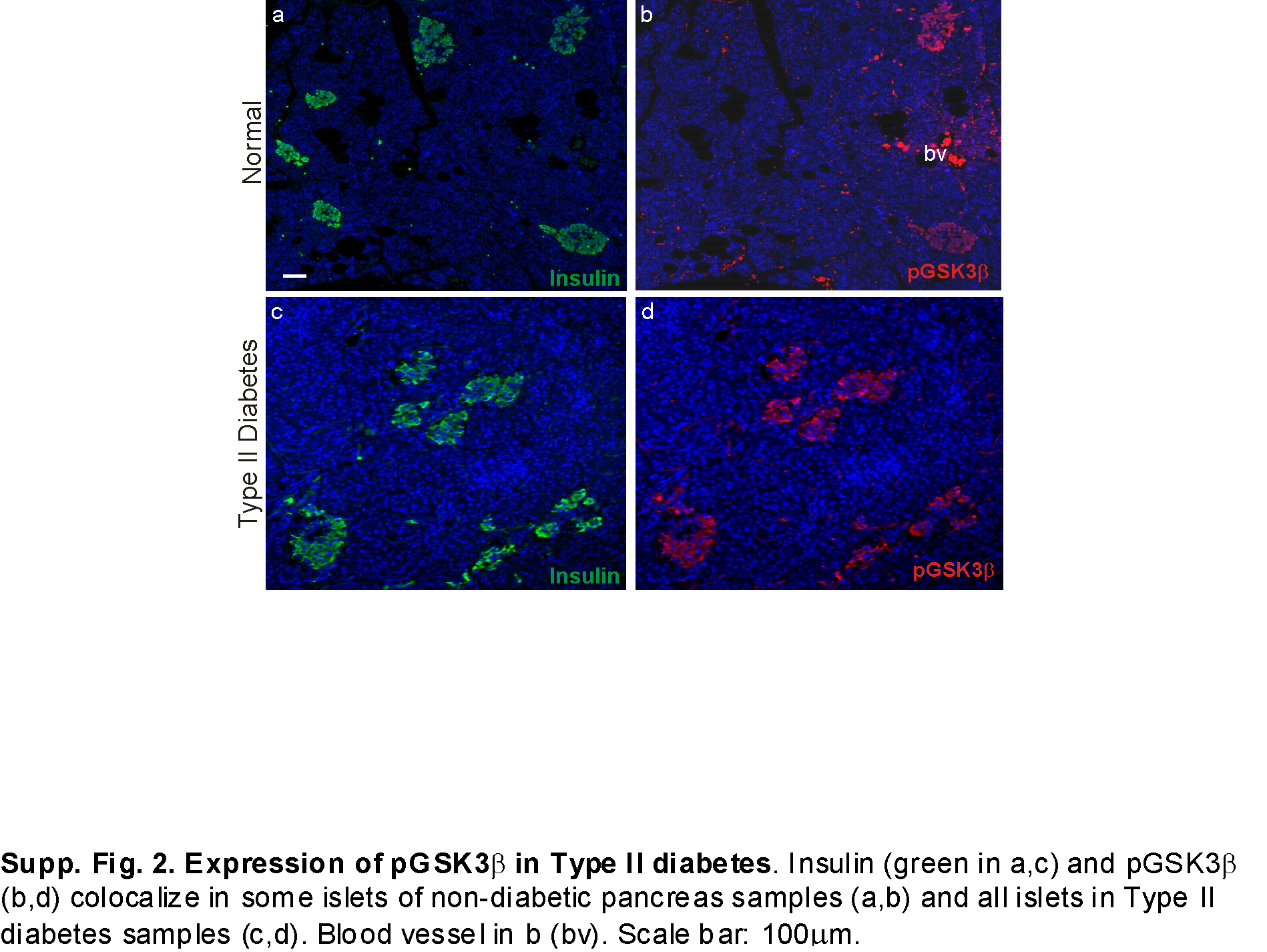

Supplement: Supplementary file 2 [file 728763.f2.tif]
